# Formulation of a Tropical Beverage by Applying Heat Treatment and High Hydrostatic Pressure

**DOI:** 10.17113/ftb.58.03.20.6459

**Published:** 2020-09

**Authors:** Carla Marengo-Orozco, Martha Patricia Tarazona-Díaz, Ligia Inés Rodríguez

**Affiliations:** Process Engineering and Industrial Systems Research Group, Department of Engineering, Jorge Tadeo Lozano University, Carrera 4 N^o^ 22-61, 110311 Bogotá, Colombia

**Keywords:** purple passion fruit, green passion fruit, carrot, high hydrostatic pressure, antioxidant capacity

## Abstract

**Research background:**

Passion fruit and carrot have a good antioxidant capacity, however, their consumption is low. There is no information on their use in beverages or in processes such as high hydrostatic pressure, which provides the safety of the drink without affecting its quality.

**Experimental approach:**

In this study the effect of high hydrostatic pressure (HHP; 500 MPa for 250 s at 25 °C) and thermal processing (at 65 °C for 10 min, 75 °C for 2 min and 95 °C for 1 min) were evaluated in the formulation of a cold-pressed beverage from purple passion fruit, green passion fruit and carrot juice, taking into account antioxidant capacity, vitamin C concentration, sensorial evaluation and microbiological growth at 8 °C.

**Results and conclusions:**

The formulation containing 67% purple passion fruit, 17% green passion fruit and 17% carrot was the one that stood out with its antioxidant capacity, high vitamin C concentration and sensorial evaluation. The HHP treatment preserved the antioxidant capacity and vitamin C concentration, and resulted in the best scent. Juices stored at 8 °C did not show microbial growth.

**Novelty and scientific contribution:**

In this study, we used tropical raw materials with good sensory acceptance and antioxidant capacity that could be used in the production of high value-added foods. Additionally, the research demonstrated that HHP is a conservation method that maintains the antioxidant capacity, vitamin C and aroma of the beverage to a greater extent compared to thermal treatments; the latter is of interest for its use in minimally processed products and functional food.

## INTRODUCTION

Fruits are a good source of antioxidants with therapeutic properties against diseases such as cancer (*1*), obesity (*2*) and Alzheimer’s (*3*), among others. Although these studies (*1*-*3*) are related to isolated substances from fruits and are conducted in cell culture or animal models, they provide insights into the possible prevention of these chronic diseases. They encourage the consumption of not only fruits and vegetables, but also of derived products such as natural juices (*4*) and their mixtures (*5*, *6*), providing new flavours and possible synergistic effects on the antioxidant capacity of such matrices (*7*, *8*).

Pasteurization is the most common method in the food industry; nevertheless, it can reduce the quality; for example, in non-centrifuged watermelon juice, pasteurization (87.7 °C, 20 s) significantly reduced the colour index, bioactive compounds such as lycopene, antioxidant capacity and total polyphenols, and the sensory quality of the juice, particularly during storage for 30 days at 8 °C. The juice was microbiologically safe for up to 30 days when stored at 4 or 8 °C (*9*). Additionally, there is a relationship between the thermal processing and the bioavailability of the bioactive compounds in the organism. For example, thermal processing could react with other compounds present in the watermelon juice, such as sugars, to form nutritionally unavailable derivatives, such as products generated in the Maillard reaction, reducing the percentage of absorption. In this way, the pasteurization (80 °C, 40 s) decreased the absorption percentage of l-citrulline in human colon cancer cells (Caco-2) (*10*). Other authors mention that thermal processing can degrade bioactive compounds (*11*, *12*) and modify organoleptic properties (*13*). For this reason, other preservation alternatives have been developed that ensure the safety of food without affecting its quality. One of them is the use of high hydrostatic pressure (HHP), which is a non-thermal method based on the application of pressure on the food matrix, able to reduce the microbiological load, preserve the organoleptic properties and bioactive compounds in the product (*14*, *15*). This technology has been used in natural products and functional food (*16*) so that the compounds of interest can reach the consumer without degrading during processing.

The present study focuses on the formulation of beverages with greater antioxidant capacity and vitamin C content from the mixture of purple passion fruit (*Passiflora edulis* f. *edulis* Sims), green passion fruit (*Passiflora maliformis*) and carrot (*Daucus carota*) because these fruits have demonstrated high antioxidant capacity (*17*–*19*). It also evaluates the effect of HHP (500 MPa, 250 s, 25 °C) with respect to three conventional thermal processings (at 65 °C for 10 min, 75 °C for 2 min, and 95 °C for 1 min). Subsequently, we carried out a sensorial analysis and microbiological follow up at 8 °C on the formulations that gave the best results to determine the best conservation method and the best formulation according to the perception of potential consumers, antioxidant capacity, vitamin C content and microbiological parameters.

## MATERIALS AND METHODS

### Chemicals and reagents

The reagents were of analytical grade, except for 96% methanol and 96% ethanol, which were of industrial grade, and provided by Ciacomeq (Bogotá, Colombia). Citric acid, 37% hydrochloric acid, l-ascorbic acid, glacial acetic acid, sodium acetate, sodium nitrite, sodium hydroxide and iron (III) chloride hexahydrate were obtained from Merk S.A. (Bogotá, Colombia). Trolox (6-hydroxy-2,5,7,8-tetramethylchroman-2-carboxylic acid), TPTZ (2,4,6-tripyridyl-s-triazine), DPPH (2,2-diphenyil-picrylhydrazyl) and 2-nitroaniline were provided by Auros Químicos Ltda (Bogotá, Colombia), and oxalic acid by Carbo Erba Reagents S.A. (Sabadell, Spain).

### Vegetal material and sample preparation

The vegetal material: purple passion fruit, green passion fruit and carrot was obtained from the local market and kept at 8 °C in the laboratory of the Jorge Tadeo Lozano University, Bogotá, Colombia, until its processing. Subsequently, the raw material was washed with potable water and disinfected with a solution of sodium hypochlorite 100 mg/L and citric acid 0.2 g/L for 5 min. Additionally, it was necessary to immerse the carrots in an anti-browning solution (ascorbic acid 10 g/L and citric acid 2 g/L). Afterwards, the juice was extracted from the obtained pulp by a cold pressing equipment (Norwalk Juicer 280; Bentonville, AR, USA).

### Effect of the preservation method on the antioxidant capacity and vitamin C content in the mixtures

An enhanced {3,2} simplex lattice design (*20*) was proposed to study the effect of the three components (juices of purple passion fruit, green passion fruit and carrot) on the antioxidant capacity and vitamin C concentration of the mixtures. First, the juices were submitted to HHP using a high-pressure processing equipment (Hiperbaric HPP, Miami, FL, USA) at 500 MPa for 250 s at 25 °C (these working conditions were defined by preliminary tests, data not shown). Pasteurisation was evaluated under three different time and temperature conditions (P_1_=65 °C for 10 min, P_2_=75 °C for 2 min and P_3_=95 °C for 1 min), with a subsequent thermal shock at 10 °C. Pasteurisation was carried out in an oil bath (Memmert, Schwabach, Germany) controlled by a thermostat by immersing the glass containers with the samples.

The response variables were the antioxidant capacity expressed in mg of Trolox equivalents (TE) per L of beverage, according to the FRAP and DPPH methods, and vitamin C concentration expressed in mg l-ascorbic acid per L of the beverage. The design consisted of 10 random combinations; 3 with only one component, 3 binary mixtures and 4 comprising all three juices (Table 1). In addition, the samples of a single component and the one that contained all the ingredients in equal amounts were done in duplicate.

**Table 1:** Enhanced simplex lattice design for the cold-pressed juice formulations

**Mixture**	***V*/mL**			**Method**	**Response variable (y)**			**Mixture**	***V*/mL**			**Method**	**Response variable (y)**		
X_1_	X_2_	X_3_	FRAP as *γ*(TE)/ (mg/L)	DPPH as *γ*(TE)/ (mg/L)	*γ*(vitamin C)/ (mg/L)	X_1_	X_2_	X_3_	FRAP as *γ*(TE)/(mg/L)	DPPH as *γ*(TE)/(mg/L)	*γ*(vitamin C)/(mg/L)
**1**	250	0	0	HHP	925.55	1491.12	323.65	5	0	125	125	HHP	647.10	842.02	82.38
					1012.71	1591.93	315.97					P_1_	719.17	1245.23	92.29
				P_1_	940.55	1445.35	172.39					P_2_	779.93	909.67	103.31
					886.02	1386.12	174.40					P_3_	560.89	756.84	74.88
				P_2_	839.99	223.73	217.33	6	125	0	125	HHP	783.04	899.93	142.93
					832.96	351.91	242.09					P_1_	701.50	1259.60	128.01
				P_3_	791.16	861.39	225.34					P_2_	927.96	619.34	159.18
					863.49	1115.76	247.99					P_3_	577.53	806.54	92.92
**2**	0	250	0	HHP	638.05	907.06	146.53	7	166	42	42	HHP	899.63	1281.69	242.79
					601.00	901.30	151.43					P_1_	855.48	1312.04	162.01
				P_1_	716.09	1370.30	136.25					P_2_	864.87	876.66	213.09
					691.12	1379.58	141.44					P_3_	692.03	989.12	166.56
				P_2_	627.91	729.16	141.18	8	42	166	42	HHP	772.85	948.58	160.96
					631.77	902.51	144.40					P_1_	779.37	1299.52	127.33
				P_3_	597.39	845.30	184.10					P_2_	783.20	806.40	152.71
					692.70	829.51	169.21					P_3_	620.16	904.36	134.31
**3**	0	0	250	HHP	360.19	446.77	18.92	9	42	42	166	HHP	709.08	860.85	48.42
					366.33	460.76	19.11					P_1_	809.01	1305.36	68.04
				P_1_	388.02	1037.03	15.18					P_2_	859.96	840.54	75.10
					388.92	971.97	17.02					P_3_	456.47	570.38	54.27
				P_2_	514.73	214.26	34.71	10	83.3	83.3	83.3	HHP	791.58	1173.52	177.30
					585.87	621.43	49.28						774.59	1202.84	163.45
				P_3_	511.98	719.79	161.24					P_1_	796.84	1242.90	128.21
					520.73	749.26	167.19						809.43	1275.64	117.38
**4**	125	125	0	HHP	812.79	1254.12	233.88					P_2_	842.27	838.11	163.94
				P_1_	774.44	1265.38	147.67						848.89	755.76	151.04
				P_2_	826.80	1046.61	210.90					P_3_	524.01	1059.78	130.68
				P_3_	752.78	857.23	199.17						542.42	972.50	127.55

X_1_=purple passion fruit juice, X_2_=green passion juice, X_3_=carrot juice. Method: high hydrostatic pressure (HHP=500 MPa for 250 s), pasteurization: P_1_=65 °C for 10 min, P_2_=75 °C for 2 min, P_3=_95 °C for 1 min. TE=Trolox equivalent

To quantify the antioxidant capacity, the sample was diluted 1:10 with 80% methanol for DPPH assay and 80% ethanol for FRAP. Afterwards, the diluted solutions were centrifuged at 2800×*g* for 15 min (centrifuge Rotofix 32; Hettich, Schwerin, Germany) and the supernatant was taken. For vitamin C quantification, the samples were treated with a 0.15% oxalic acid solution in sufficient quantity according to Bernal de Ramírez (*21*) to provide a concentration between 0.04 and 4 mg l-ascorbic acid per mL. Then, the samples were centrifuged at 2800×*g* for 15 min (Rotofix 32; Hettich) and the supernatant was taken.

#### Determination of the antioxidant capacity by FRAP assay

Ferric reducing antioxidant power (FRAP) assay is useful for the determination of antioxidants present in a liquid sample. It is based on the reduction of the iron (III)-tripyridyltriazine to iron(II)-tripyridyltriazine with antioxidants (reducing agents) in acidic solution. This assay was carried out in triplicate according to the method described by Benzie and Strain (*22*) as follows: 30 µL of diluted extract were mixed with 30 µL of 80% ethanol and 940 µL of FRAP reagent (composed of 0.3 mol/L buffer sodium acetate pH=3.6, 0.01 mol/L tripyridyltriazine and 0.02 mol/L iron(II) chloride (10:1:1)). Then, the sample was incubated at 37 °C for 1 h in the absence of light. Subsequently, the absorbance was measured at 593 nm in the spectrophotometer (Evolution™ 300; Thermo Fisher Scientific, Loughborough, UK) and it was compared with a Trolox standard linear curve between 100 and 600 µmol/L. The antioxidant capacity was expressed as Trolox equivalent in mg/L.

#### Determination of antioxidant capacity by DPPH assay

This method is based on the reduction of stable DPPH nitrogen radicals in the presence of antioxidants. Antioxidant capacity was determined according to the method described by Brand-Williams*et al.* (*23*) in triplicate. A volume of 50 µL of diluted extract, 800 µL of 80% methanol and 200 µL of methanol solution of 6·10^-4^ mol/L DPPH were mixed. Then, the mixture was incubated at 37 °C for 1 h in the absence of light. The absorbance was measured at 515 nm (Evolution™ 300; Thermo Fisher Scientific), and compared to a Trolox calibration curve in the concentration range from 100 to 600 µmol/L. The results were expressed as Trolox equivalents in mg/L.

#### Determination of vitamin C using 2-nitroaniline colourimetric method

The l-ascorbic acid is treated with diazotized 2-nitroaniline diazotized in the presence of an excess of NaOH, which turns to 2-nitrophenylhydrazine gives a red-violet sodium salt solution with absorption maximum at 540 nm. Vitamin C was determined according to the method described by Bernal de Ramírez (*21*) in triplicate. For this assay, the components were added in the following order: 10 µL of 2-nitroaniline reagent, 20 µL of sodium nitrite, 380 µL of 96% ethanol, 50 µL of diluted extract, 50 µL of 0.15% oxalic acid, 120 µL of 10% NaOH and 380 µL of distilled water. The absorbance was then measured at 540 nm (Evolution™ 300; Thermo Fisher Scientific). It was compared to l-ascorbic acid calibration curve in the concentration range from 20 to 200 mg/L. The results were expressed in mg/L of l-ascorbic acid.

### Sensory and microbiological analyses

The formulations containing the three ingredients (mixtures 7, 8, 9 and 10 in Table 1) were subjected to the four conservation methods (HHP, P_1_, P_2_ and P_3_). These samples were taken for sensory evaluation of colour, aroma, taste and general acceptance. The test was conducted with an informal sensory panel consisting of 30 people (15 women, 15 men, aged between 20 and 28). The tests were carried out with the products at 20 °C using a seven-point hedonic scale where 7=like a lot, 5=like a little, 4=dislike, 3=dislike a little, and 1=dislike it a lot with a marketing limit set at 5.

The mixtures containing the three raw materials according to the enhanced simplex lattice design (Table 1) were subjected to the four conservation methods and then stored at (8±2) °C. Every 15 days, during 60 days, total mesophilic aerobic bacteria were counted on plate count agar, and moulds and yeasts on potato dextrose agar. The results were expressed as colony forming units per mL. The microbiological parameters were determined in triplicate.

### Statistical analysis

The response surface methodology was used to determine the effect of conservation methods on antioxidant capacity and vitamin C content in the formulations. The experimental design was carried out with program Design-Expert, v. 9.0 (*24*). The choice of the response function that best fitted the experimental data was made using ANOVA (p<0.05), considering the linear, quadratic, cubic and special quartic models. After the analysis of the model, the associated contour graphs were generated, and the best mixing conditions were defined.

To determine the effect of conservation methods and the formulation on the sensorial evaluation, the Kruskal Wallis (p<0.05) test was carried out with the statistical program Infostat, v. 2018e (*25*), performing the Mann-Whitney U test (p<0.0083) in the case of the occurrence of a significant difference.

## RESULTS AND DISCUSSION

### Antioxidant capacity and vitamin C concentration in the mixtures

#### FRAP assay

**Fig. 1:**
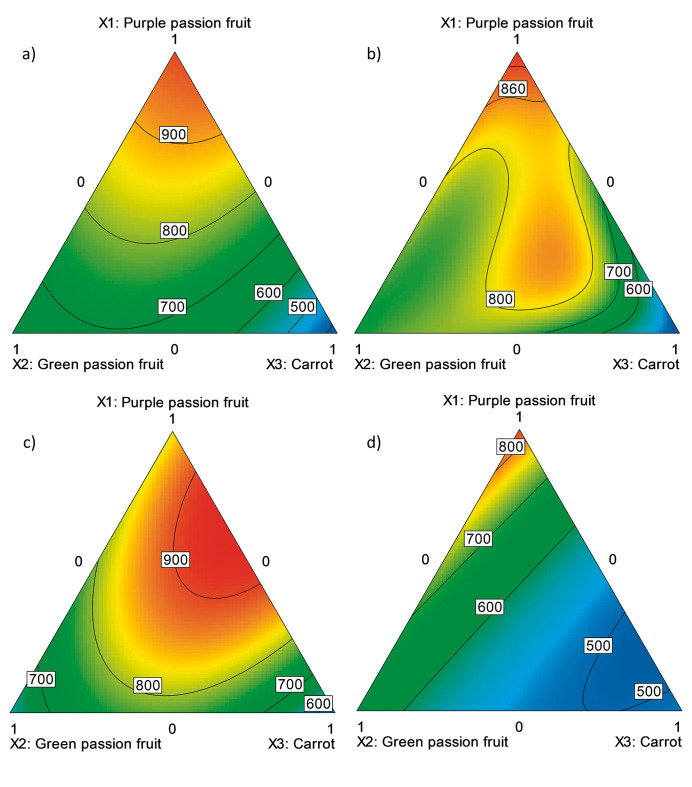
The effect of the formulation of a drink made from purple passion fruit juice, green passion fruit juice and carrot juice treated by: a) high hydrostatic pressure (500 MPa, 250 s, 25 °C), pasteurisation: b) P_1_ (65 °C, 10 min), c) P_2_ (75 °C, 2 min) and d) P_3_ (95 °C, 1 min) on the antioxidant capacity determined by FRAP method (expressed as Trolox equivalent in mg/L)

Regression analysis of the results obtained for the determination of antioxidant capacity with FRAP assay (Table 1), shows good linearity with high coefficients of determination (Table 2) (R^2^_adj_≥0.86 and model with p≤0.0014 in test F), which implied that the variability in the antioxidant capacity of the beverage is explained to a high degree by its components. Additionally, according to the contour graphs (Fig. 1), component X_1_ (purple passion fruit) is the largest contributor to the antioxidant capacity, while formulations with a high carrot content (component X_3_) had a lower value in the response variable. Nevertheless, according to Fig. 1c, there is a synergistic effect between these components. Fig. 1a also shows a similar behaviour of the green passion fruit (X_2_) and carrot (X_3_) because the mixtures with high content of these raw materials have a greater antioxidant capacity than what has been reported for the pure juices from these fruits.

**Table 2:** Regression coefficients for the models of antioxidant capacity and l-ascorbic acid determination in juices treated with high hydrostatic pressure (HHP) and pasteurisation (P) method

**Parameter**	**FRAP as *γ*(TE)/(mg/L)**				**DPPH as *γ*(TE)/(mg/L)**				**Vitamin C as *γ*(l-ascorbic acid)/(mg/L)**			
HHP	P_1_	P_2_	P_3_	HHP	P_1_	P_2_	P_3_	HHP	P_1_	P_2_	P_3_
**Model**	***	**	***	***	***	***	*	*	***	***	***	-
**Linear mixture**	***	***	***	***	***	***	*	*	***	***	***	-
**Purple passion fruit juice** **X_1_**	966.18	916.50	833.16	834.85	1538.32	1410.78	317.16	1011.47	319.11	175.16	244.38	236.95
**Green passion fruit juice** **X_2_**	621.58	706.81	631.74	651.78	900.98	1370.83	796.17	863.64	148.28	139.00	151.59	176.04
**Carrot juice X_3_**	369.82	391.68	562.14	514.18	450.56	1019.14	442.36	704.40	18.32	14.57	45.09	157.82
**X_1_X_2_**	100.44	-97.51	237.13	-58.71	86.59	-535.91*	1637.80*	-	-10.44	-33.49	-	31.83
**X_1_X_3_**	521.01**	240.96	860.45***	-555.71*	-429.33	294.34	989.74	-	-114.32*	123.22**	-	-403.00
**X_2_X_3_**	706.55**	731.04*	712.95**	-262.57	613.73*	323.49	801.01	-	-14.87	39.79	-	-360.48
**X_1_^2^ X_2_X_3_**	-	-3146.14	-	-	5281.24	-	-	-	2217.07	-	-	-
**X_1_X_2_^2^X_3_**	-	-4454.30	-	-	-5665.84	-	-	-	1708.76	-	-	-
**X_1_X_2_X_3_^2^**	-	12717.89*	-	-	13825.18*	-	-	-	-2492.74*	-	-	-
**R^2^**	0.98	0.98	0.95	0.91	0.99	0.91	0.77	0.44	1.00	0.99	0.95	0.93
**R^2^_adj_**	0.96	0.94	0.91	0.86	0.98	0.85	0.62	0.34	0.99	0.98	0.94	0.86

Conservation method: HPP=500 MPa, 250 s, 25 °C, P_1_=65 °C, 10 min, P_2_=75 °C, 2 min, P_3_=95 °C, 1 min  *Significant at 0.05; **significant at 0.01; ***significant at 0.001. TE=Trolox equivalent

The conservation methods also affected the antioxidant capacity of the formulations. Treatments with HHP and P_2_ gave the highest values (Fig. 1a and 1c), while the method P_3_ (Fig. 1d) showed the lowest results in the trial, which could be explained by the fact that the high temperatures of the process can degrade some antioxidant compounds (*11*, *12*). On the other hand, sample 6, which was subjected to P_2_ method, had the highest antioxidant capacity expressed as Trolox equivalents (927.96 mg/L; Table 1). The above demonstrates the potential of the beverage with respect to other products on the market, with higher values than blueberry juice type “Toro” (0.77±0.01) mg/g) obtained by Kraujalytė *et al.* (*26*), and Veltlín zelený white wine ((535.62±2.50) mg/L), but lower than Modrý Portugal red wine ((1231.43±2.50) mg/L) characterized by Stratil *et al.* (*27*). In addition, the antioxidant capacity of the beverage was lower than that obtained by Abountiolas and do Nascimento Nunes (*28*) for the pomegranate juice Lakewood Pure Pomegranate (Lakewood Juices, Inc.) (8214.01 mg/L), but higher than that of orange juice Minute Maid Orange Juice (Coca Cola Company) (509.70 mg/L).

#### Results of DPPH assay

**Fig. 2:**
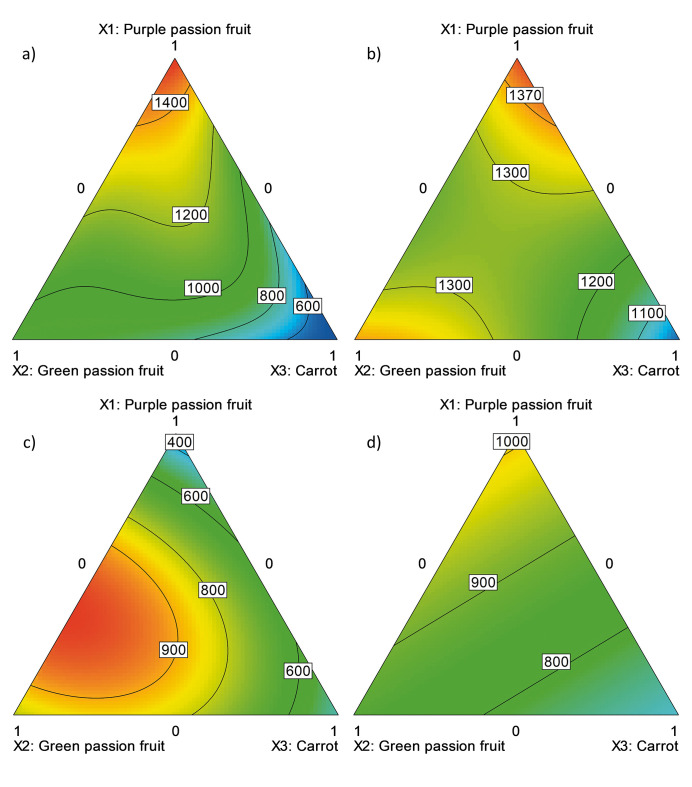
The effect of the formulation of a drink made from purple passion fruit juice, green passion fruit juice and carrot juice treated by: a) high hydrostatic pressure (500 MPa, 250 s, 25 °C), pasteurisation: b) P_1_ (65 °C, 10 min), c) P_2_ (75 °C, 2 min) and d) P_3_ (95 °C, 1 min) on the antioxidant capacity determined by DPPH method (expressed as Trolox equivalent in mg/L)

Only two of the models achieved coefficients of determination higher than 0.8 (Table 2). Nevertheless, although it is not possible to determine the effect of the formulation on the antioxidant capacity of the beverage treated with methods P_2_ and P_3_, it is observed that the antioxidant capacity decreased for all the models of the experimental design, which allows inferring that the increase in temperature affects the antioxidant capacity of the mixtures, generating models and contour graphs with values lower than those obtained in HHP and P_1_ treatments (Fig. 2). **FIGURE 2**

According to Figs. 2a and 2b, the juice of purple passion fruit is the raw material that provides the greatest antioxidant capacity, while the carrot juice has the lowest values. These results are similar to those of the FRAP assay.

On the other hand, mixture 7 was the one which showed the highest antioxidant capacity (Table 1), since it contains the highest volume fraction of purple passion fruit juice. This quantification exceeds the antioxidant capacity determined for untreated pineapple juice ((1096±193) mg/L) (*29*) and white wines ((445.52±7.51) mg/L), similar to that of red wine ((1379.1±2.5) mg/L) (*27*). In addition, the antioxidant capacity of the formulation exceeds the one determined for the blueberry and raspberry juice Fuze Slenderize Blueberry and Raspberry (Coca Cola Company) (814.94 mg/L), while it is lower than the pomegranate-based drinks (7854.83 mg/L) Lakewood Pure Pomegranate (Lakewood Juices, Inc.) (*28*).

#### Determination of vitamin C concentration

**Fig. 3:**
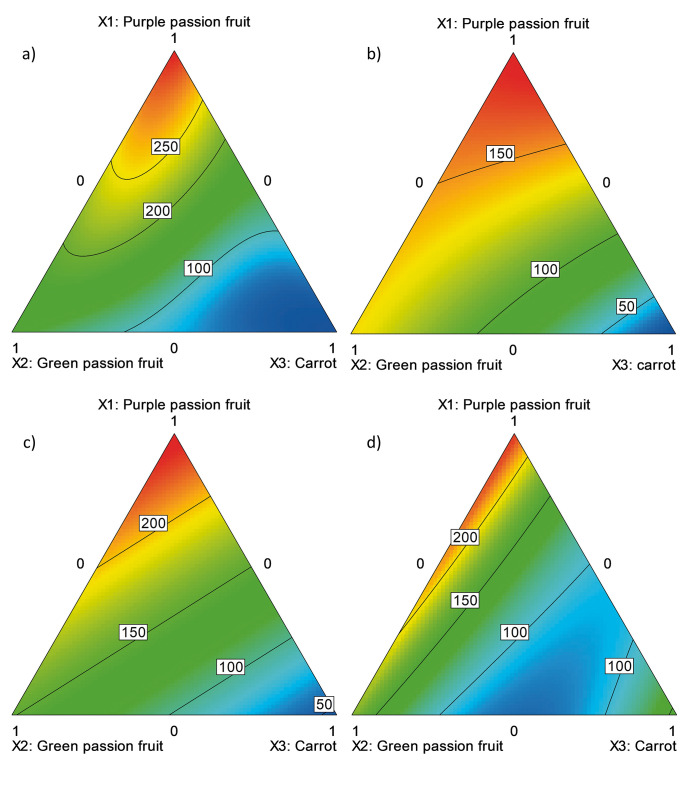
The effect of the formulation of a drink made from purple passion fruit juice, green passion fruit juice and carrot juice treated by: a) high hydrostatic pressure (500 MPa, 250 s, 25 °C), pasteurisation: b) P_1_ (65 °C, 10 min), c) P_2_ (75 °C, 2 min) and d) P_3_ (95 °C, 1 min) on the l-ascorbic acid content (expressed as l-ascorbic acid in mg/L)

With the obtained results (Table 1), the statistical models with the best fit (Table 2) were generated (R^2^_adj_≥0.86 and model with p≤0.002 in the F test) and the respective contour graphs (Fig. 3) were plotted. From the above, it is possible to affirm that, using all conservation methods, purple passion fruit juice contributes to the formulation with the greatest concentration of l-ascorbic acid, followed by green passion fruit juice and finally carrot juice. Additionally, none of the graphs show possible synergistic effects among components. Nevertheless, according to Fig. 3d, an antagonistic effect between green passion fruit and carrot is observable, which is especially noted when there is a low volume fraction of purple passion fruit in the mixture.

The concentration of l-ascorbic acid in the formulations was also affected by the conservation process, and the methods which showed higher vitamin C concentration in the decreasing order were HHP and P_2_ (Figs. 3a and 3c). The results confirm the degradation of ascorbic acid during thermal treatments, as reported by some authors (*13*, *30*), which is notable in P_3_ method compared to HPP (Figs. 3a and 3d) at 25 °C. On the other hand, prolonged times of exposure to high temperatures show decreases in the vitamin C concentration, as shown in Fig. 3b, where values are lower than in P_2_ (Fig. 3c), in spite of the fact that the latter method had a higher temperature for a shorter time interval.

The highest concentration of l-ascorbic acid (0.24 mg/g) was determined in mixture 7, which was subjected to HPP (Table 1), similar to the results of Rao *et al.* (*30*) for peach juice pasteurized at 90 °C for 1 min ((263.4±9.5) mg/L), or those for fresh purple passion fruit juice (0.29 mg/g) (*31*). However, the vitamin C concentration of the formulation is lower than what was described for fresh orange juice (536.5 mg/L) (*13*) and for beverage R.W. Knudsen Mega C (Knudsen & Sons, Inc.) (4606 mg /L) (*28*).

### Sensory analysis

According to Table 3, mixtures 7, 8 and 10 exceeded the marketing limit established for all the evaluated characteristics (*5*). On the contrary, mixture 9 showed the lowest values for the parameters: colour, aroma and general acceptance, while it did not demonstrate significant differences in the flavour in relation to mixtures 7 and 8. The above shows that the formulations with low carrot content are preferred by the sensory panel.

**Table 3:** Sensory evaluation of the selected cold-pressed juice formulations

**Parameter**	**Mixture**			
7	8	9	10
**Colour**	(5.4±1.1)^a^	(5.6±1.0)^a^	(5.0±1.3)^b^	(5.6±1.0)^a^
**Aroma**	(5.3±1.0)^ab^	(5.2±1.1)^b^	(4.45±1.2)^c^	(5.6±0.9)^a^
**Flavour**	(5.0±1.2)^b^	(5.0±1.1)^b^	(4.6±1.4)^b^	(5.5±1.2)^a^
**General acceptance**	(5.3±0.8)^b^	(5.3±0.8)^b^	(4.85±0.9)^c^	(5.6±0.8)^a^

The same letters in a row denote no significant differences based on a Kruskal Wallis test (p<0.05) and Mann-Whitney U test (p<0.0083)

No significant differences were observed in the colour (5.4±1.1), flavour (5.0±1.2) and general acceptance (5.2±0.8) characteristics, with respect to the applied conservation method. The above did not follow the behaviour reported by Zvaigzne *et al.* (*13*), who showed that high temperature pasteurization and short time (130 °C for 2 s) affect to a lesser extent the sensorial characteristics of orange juice than conventional pasteurization (94 °C for 30 s). On the other hand, the aroma showed changes depending the preservation treatments, where the HHP treatment resulted in a significantly higher values (5.5±1.2) than treatments P_2_ (5.0±0.9) and P_3_ (4.9±1.0), which is similar to the findings of Rao *et al.* (*30*) in peach juice, where the samples treated with HHP (600 MPa, 10 min, 25 °C) were better evaluated than the pasteurized beverage (90±2) °C, 1 min). For this reason, it is inferred that the HHP method keeps the aroma of beverages to a greater extent than thermal treatments.

### Microbiological assay during storage at 8 °C

All the mixtures submitted to the four conservation treatments showed a concentration of <10 CFU/mL of mesophilic aerobes, as well as moulds and yeasts on day 0 (data not shown). The thermally treated samples showed an increase in the concentration of mesophilic aerobes at 15 days (30-350 CFU/mL), being uncountable after 30 days. There was no evidence of mould and yeast growth during the first 15 days of storage, and after 30 days they were uncountable.

Juices submitted to HHP exhibited a maximum concentration of 15 CFU/mL during the 60 days of experimentation, showing a null growth of microorganisms during storage. The above is similar to what was reported for pomegranate juice, where treatments with HHP higher than 350 MPa for 150 s maintained the mesophilic aerobic parameters, moluds and yeasts below the detection levels during the entire storage period (<1.0 log CFU/mL during 35 days) (*32*). Nevertheless, Patterson *et al.* (*33*) further explained that although the use of HHP (500-600 MPa, 1 min, 20 °C) reduces the microbial population to 4 log CFU/mL in carrot juice, the population of microorganisms at 8 °C increases during storage, with strains of lactic acid bacteria and Gram-positive spore-forming bacteria determined in carrot juice.

## CONCLUSIONS

The mixture composed of purple passion fruit (67%), green passion fruit (17%) and carrot (17%) juices treated at 500 MPa and 25 ºC for 250 s showed the highest antioxidant capacity expressed as Trolox equivalent (899.63 and 1281.69 mg/L by FRAP and DPPH assay, respectively), vitamin C concentration (242.79 mg/L) and sensory acceptance. The thermal treatments affected the antioxidant capacity and the concentration of vitamin C in the mixtures, while the high hydrostatic pressure preserved these components and demonstrated better results of the aroma evaluation of the formulations and microbial control during storage at 8 °C. For this reason, high hydrostatic pressure presents an emerging technology that does not affect the analysed chemical parameters, preserves the organoleptic properties desired by the consumers, and ensures the harmlessness and extends the shelf life of the food.
